# Design and clinical implementation of a TG‐106 compliant linear accelerator data management system and MU calculator

**DOI:** 10.1120/jacmp.v11i3.3212

**Published:** 2010-04-30

**Authors:** Nabil Adnani

**Affiliations:** ^1^ Chief Products Development Officer, D3 Products Division Pittsburgh PA 15206 USA

**Keywords:** linac commissioning, data book, beam modeling, TG‐51, calibration, MU calculator, quality assurance, treatment planning

## Abstract

In an attempt to minimize errors and improve patient outcome in radiation therapy, a linear accelerator data management system was developed to provide radiation oncology physicists with a set of computerized tools to manage linear accelerator physics data. The entire program is written in Microsoft Visual Basic and has a user‐friendly, front‐end window with the following features and modules: (1) Generate, edit and approve commissioning and QA reports and other regulatory documents, (2) Configure commissioning tasks, (3) Acquire output factors, (4) Import scanned data, (5) Import PDD, TMRs and OAR tables directly from the scanning software, (6) Query physics data such as TMR, PDDs, OFs, and WFs, (7) Compare physics data to a different machine or a standard, (8) Compare physics data from the same machine (e.g. during annual calibrations), (9) Perform MU calculations on plans exported from the planning system via DICOM RT, (10) Perform TG‐51 calibration, (11) Perform monthly calibration, (12) FTP physics data for purposes of remote peer review and/or inspections.

PACS numbers: 87.53.Dq, 87.55.Qr, 87.56.Fc

## I. INTRODUCTION

The process of commissioning a linear accelerator requires, among other tasks, the acquisition and processing of a significantly large amount of physics data. This data is later used to calculate the dose delivered to patients about to undergo radiation treatments.^(^ ^1^ ^–^ ^2^ ^)^ In most cases, commissioning is performed only once in the lifetime of the machine. The volume of measurements involved is so large, it is no surprise that the entire process is considered one of the most complex and error‐prone in radiation oncology today.^(^ ^3^ ^)^ Recently, the Radiological Physics Center of MD Anderson (Houston, Texas) conducted a credentialing study of radiation oncology centers throughout the USA. Their results showed an alarming number of institutions failing to pass clinically acceptable tolerance limits of 7% dose difference and 4 mm distance to agreement.^(^ ^4^ ^)^ Their phantom irradiations, as part of the credentialing efforts, identified the following errors:

1. Incorrect output factors and percentage depth dose data.
2. Inadequate modeling of the penumbra at multileaf collimator leaf ends.
3. Incorrect application of QA calculations or measurements.
4. Inadequate QA of multileaf collimator.
5. Incorrect patient positioning, including couch indexing errors with serial tomography system.
6. Errors in treatment planning software.

Very much aware of this reality, physicists are always striving to improve their processes in an effort to minimize errors in dose delivery.

## II. MATERIALS AND METHODS

### A. Software system

One possible solution to minimizing errors in physics data, considered in this report, would be to reduce, as much as possible, manual handling and processing which, in addition to setup errors, is usually considered the most likely source of error. To this end, a software application has been developed with the following general features:

1. Provide a computerized system by which all conventional methods of physics data processing, using Microsoft Excel or similar means, are performed.
2. Provide tools for comparing newly acquired data to existing machine data that is properly validated.
3. Provide tools to manage the data acquisition process.
4. Provide tools to validate beam models generated by treatment planning systems using the newly acquired physics data.

**Figure 1:**
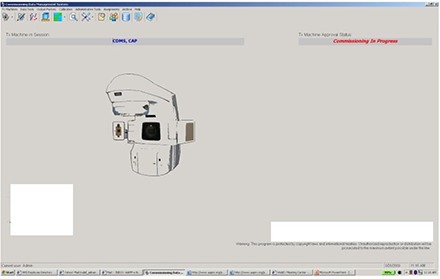
The main user interface through which all of the system's features are accessed.

The software is a Windows application, referred to as Comprehensive Data Management Suite or CDMS. It has an easy to use GUI (see Fig. 1) allowing users to access all of the features and modules of the system, which can be installed as a standalone application or on a network server.

#### A.1 CDMS commissioning goals

1. Simplify data acquisition.
2. Minimize errors in collected data.
3. Provide beam modeling and calibration tools.
4. Generate data books (eData Books or in print format) and commissioning reports.
5. Standardize data acquisition, data access and corresponding documentation and reports.

#### A.2 CDMS Clinical Goals

1. Improve patients' outcome through data errors minimization.
2. Perform MU calculations.
3. Perform monthly and annual calibration and generate corresponding reports.
4. Simplify physics audits, peer review and credentialing efforts.

### B. System design characteristics

**Figure 2:**
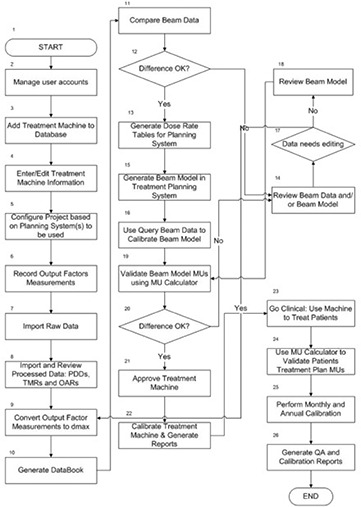
Processes in the lifetime of a linac currently supported by CDMS.

**Figure 3:**
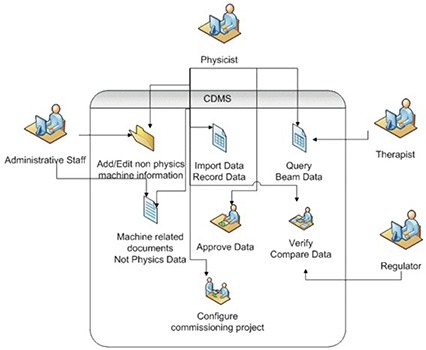
Example of CDMS users together with their most accessible features.

Figure 2 shows a flowchart diagram of the commissioning and clinical processes managed by CDMS. The system can be used by administrative as well as clinical staff. Figure 3 gives an example of CDMS users, together with some of their commonly accessed features. All of the steps required to acquire and document the physics data as recommended by AAPM Task Group No.106^(^ ^5^ ^)^ are organized in a series of modules. These modules are divided into three categories:

#### B.1 Data management

The data management category of tools includes modules to create a new treatment machine, or to edit or import an existing treatment machine. PDD, TMR and OAR data tables generated by third‐party scanning software can also be imported. So far, CDMS is compatible with data tables generated by OmniPro from Wellhofer (IBA Group, Bartlett, TN) and MEPHYSTO mc^2^ from PTW (PTW, NY). Also, within the same category, tools are available to help physicists configure their commissioning projects well in advance of starting actual measurements. In addition, all associated documentation, such as calibration and commissioning reports, can be automatically generated. Finally, communication tools are provided to transfer entire treatment machine data to a remote location.

#### B.2 Data acquisition

With this category of tools, common errors originating from manual data entry and/or processing are minimized by allowing chamber readings, taken during output factor measurements, to be recorded and organized by beam type, energy and accessory. These are then immediately available for calculations purposes, data book generation and beam modeling needs.

#### B.3 MU calculation

The presence within CDMS of all relevant beam dosimetry data makes it possible for MU calculations to be performed either manually or using CDMS' DICOM RT import filter. The Query Beam Data, as its name indicates, allows for an electronic query of common treatment field parameters such as TMRs, PDDs, OARs, OFs and TFs. It also provides the user with the option to perform MU calculations for a given prescribed dose in the absence of a treatment plan. The MU calculation tool uses a DICOM RT filter to import plans directly from a treatment planning system. By selecting the desired plan, a list of available fields is displayed and MU calculations are performed and compared to the planning system's MUs. A report is also automatically generated for documentation and review purposes.

### C. Clinical Implementation

CDMS is currently the standard for linac commissioning and beam modeling for our practice. Our physicists rely entirely on CDMS during the preparation stages and actual measurements. Once the measurements phase is completed and all required data available, CDMS helps with beam modeling, data book generation and linac calibration.

**Figure 4:**
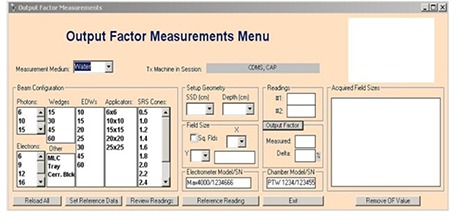
Output factors measurements menu.

Since its deployment in 2008, more than 20 Varian linacs were commissioned by CDMS. The process begins by creating the new treatment machine. Administrative and technical details (see Fig. 4), the associated treatment planning system, the water phantom, ionization chamber and electrometer to be used for measuring output factors are all entered at this stage. Once the treatment machine is created, all of the physics parameters needed to properly commission the machine are entered using the “Configure Project”. These include the SSDs, depths and required field sizes for output factor measurements.

#### C.1 Output factors measurements

**Figure 5:**
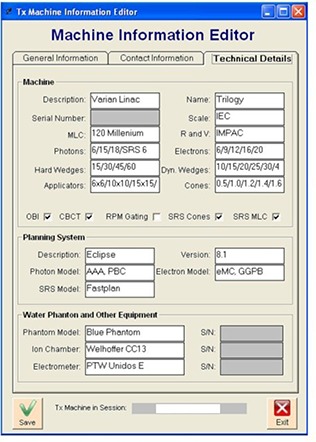
Administrative and technical details menu.

The output factor measurements menu, shown in Fig. 5, is used to acquire OF and organize them in the treatment machine database by radiation type, energy and accessory. Details about the measurement geometry, SSD and depth are also recorded. These are later used to convert measurements at a given geometry to the corresponding $d_{\max}$ values as follows:

(1) $$OF_{d\;\text{max}}(\text{Field}\;\text{Size}\;X)=OF_{depth}(\text{Field}\;\text{Size}\;X)\times \frac{TMR_{depth}(\text{Reference}\;\text{Field}\;\text{Size})}{TMR_{depth}(\text{Field}\;\text{Size}\;X)}$$

(2) $$\approx OF_{depth}(\text{Field}\;\text{Size}\;X)\times \frac{PDD_{depth}(\text{Reference}\;\text{Field}\;\text{Size})}{PDD_{depth}\;(\text{Field}\;\text{Size}\;X)}$$

A delta difference expressed as a percentage difference between acquired and expected value for a given beam and setup geometry is provided throughout the measurements session to provide guidance to the commissioning physicists and, at the same time, minimize measurement errors resulting from detector, phantom or treatment machine.

#### C.2 Import beam scans and compare treatment machine data

PDDs, TMRs and OARs tables are generated by third‐party software used for beam scanning. These are then imported to CDMS (see Fig. 5). In CDMS, these tables can be electronically queried, displayed in a clinically friendly format, or simply printed as part of the treatment machine data book.

Common beam dosimetry parameters such as PDDs, TMRs, OARs, OFs, and WFs between two different machines or the same machine scanned at different times, as is the case during annual calibration,^(^ ^6^ ^)^ are compared using the “Compare Beam Data” tool.

#### C.3 TG‐51 calibration and dose rate tables

TG‐51 calibration and report generation is made easy through CDMS. All TG‐51 chamber and electrometer parameters such as $P_{\text{ion}}$ and $P_{\text{pol}}$ are stored in the treatment machine's database. These are then used on a monthly basis to perform routine linac calibrations. TG‐51 and monthly reports are generated electronically immediately following the calibration session. Reports are organized in menus allowing for easy review, verification and approval. Approved reports cannot be edited.

Another source of errors during commissioning is the generation of the dose rate tables from processed measured output factors tables. CDMS provides tools to generate these tables in a format that is required by the treatment planning system. In the case of Eclipse (Varian Medical Systems, Palo Alto California), a choice between AAA and PBC algorithms is provided.

#### C.4 MU calculations and beam model verification

**Figure 6:**
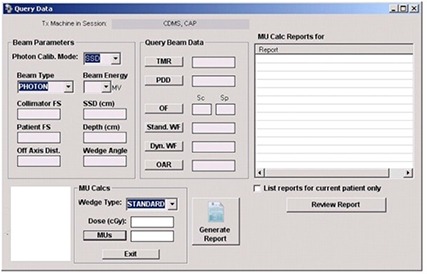
The query beam data module.

**Figure 7:**
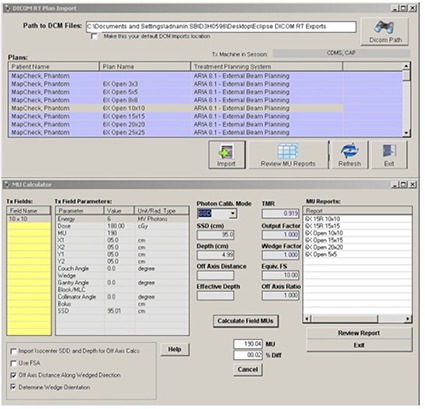
DICOM RT enabled MU calculator.

CDMS offers the options of performing MU calculations, for both photons and electrons, either by manually entering the beam parameters (Fig. 6) or by importing the entire treatment plan using its DICOM RT import filter (Fig. 7).

##### C.4.1 MU calculator algorithm

CDMS uses the physics data either imported to, or generated within, CDMS to derive its own MUs using Khan's algorithm^(^ ^7^ ^)^ for both photons and electrons.

##### C.4.2 Wedge beam hardening effect

**Figure 8:**
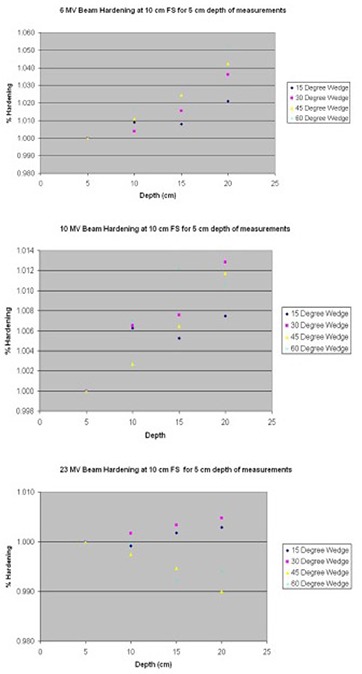
Effect of beam hardening on wedge transmission factor. The examples of 6 MV, 10 MV and 23 MV are shown when transmission factors are measured at 5 cm depth.

Since CDMS MU calculator uses the open field TMRs, beam hardening resulting from the use of physical wedges needs to be taken into account.^(^ ^8^ ^)^ Figure 8 shows the percent increase in wedge transmission factors as a function of depth at 10cm×10cm field size for 6 MV, 10 MV and 23 MV when wedge transmission factors are measured at 5 cm depth. These variations are converted to a functional fit and used by CDMS to include the effect of beam hardening away from the depth of measurement.

##### C.4.3 Heterogeneity corrections

The equivalent path length is used to account for heterogeneity corrections.^(^ ^7^ ^)^ When the calculation point is within the heterogeneity itself, a field size scaling factor is used as follows:

(3) $$FS_{Adjusted}=FS_{Actual}\times \frac{Depth_{effective}}{Depth_{physical}}$$

##### C.4.4 Validation of the MU calculator

A combination of both calculations and measurements were performed to validate the MU calculator in CDMS. A total of 76 plans were generated combining open and wedged field (physical and dynamic) using the Eclipse treatment planning system (Varian Medical Systems, Palo Alto, CA). The plans are then exported using DICOM RT to CDMS for MU calculations. The same plans were delivered at the machine and dose, at the point of calculations, was measured using MapCHECK (Sun Nuclear, Melbourne Florida).

Prior to going clinical, the DICOM RT MU calculations module helps during the preliminary validation (or verification) of beam models and the documentation of the corresponding results in the generated reports. It is important to note that the use of the MU calculator during beam model verification is only useful for identifying gross errors in the beam model. An example of such error would be to enter PDD curves for 10 MV instead of 6 MV. This step, however, does not constitute a full commissioning of the planning system, which requires validation by measurements, as recommended by TG‐53.^(^ ^9^ ^)^

#### C.5 Document manager and data transfer

**Figure 9:**
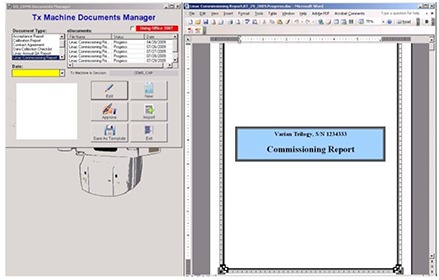
Documents manager module.

One of the main recommendations of TG‐106^(^ ^5^ ^)^ is the proper documentation of a commissioning project by generating an appropriate final report. CDMS is designed to help physicist automatically populate a commissioning report template with all of the physics data of the treatment machine. The ionization chamber and electrometer calibration factors used during TG‐51 calibration also automatically populate the commissioning report template. Figure 9 shows a screen shot of the document manager and a sample linac commissioning report thus generated. Documents can be electronically approved through CDMS Documents Manager. An approved document cannot be edited.

All of the physics data and associated documentation of a given treatment machine can be sent to a specific location via FTP. This tool is currently heavily used by our practice to organize the assignment of commissioning jobs to a physicist in the field, as well as to upload a completed or partially completed job back to our practice. The ability to transfer the entire linear accelerator physics data from one location to another may prove to be very useful for remote peer review,^(^ ^10^ ^)^ as well as for state inspection purposes.

## III. RESULTS

### A. MU calculator validation

Tables 1 and 2 give a cross section of the comparison between Eclipse‐calculated MUs, and those generated by CDMS for the same treatment plan. It also validates both Eclipse and CDMS by measuring the dose delivered at the calculation point compared to the one prescribed by the plan to the same point.

**Table 1:** Open fields central axis CDMS MU calculator validation.

*Energy (MV)*	*Field Size (cm)*	*Depth*	*TMR*	*OF*	*Inv. Sq.*	*Eclipse MUs*	*CDMS MUs*	*MU %Diff*	*Eclipse Dose (cGy)*	*Measured Dose (cGy)*	*Dose %Diff*
	5×5	5 cm	0.898	0.948	1.03	207.00	205.25	0.85	180.00	179.44	−0.32
	8×8	5 cm	0.914	0.981	1.03	195.00	194.72	0.14	180.00	181.90	1.04
	10×10	5 cm	0.920	1.000	1.03	190.00	189.92	0.04	180.00	180.81	0.43
6	15×15	5 cm	0.930	1.033	1.03	183.00	182.68	0.17	180.00	180.32	0.13
	20×20	5 cm	0.936	1.047	1.03	179.00	178.37	0.35	180.00	178.90	−0.63
	25×25	5 cm	0.940	1.05	1.03	175.00	175.72	0.41	180.00	176.75	−1.82
	30×30	5 cm	0.943	1.073	1.03	173.00	172.65	0.20	180.00	177.21	−1.55
	5×5	5 cm	0.998	0.926	1.067	182.00	182.46	0.25	180.00	179.88	−0.08
	8×8	5 cm	0.996	0.979	1.067	173.00	173.00	0.00	180.00	179.23	−0.43
	10×10	5 cm	0.993	1.000	1.067	170.00	169.87	0.08	180.00	179.60	−0.22
23	15×15	5 cm	0.986	1.040	1.067	165.00	164.37	0.38	180.00	179.35	−0.37
	20×20	5 cm	0.983	1.063	1.067	161.00	161.49	0.30	180.00	177.45	−1.42
	25×25	5 cm	0.982	1.077	1.067	159.00	159.40	0.25	180.00	176.86	−1.74
	30×30	5 cm	0.981	1.092	1.067	158.00	157.47	0.34	180.00	178.79	−0.67

**Table 2:** Wedged fields central axis CDMS MU calculator validation.

*Wedge Angle*	*Energy (MV)*	*Field Size (cm)*	*TMR*	*WF*	*OF*	*Inv. Sq.*	*Eclipse MUs*	*CDMS MUs*	*MU %Diff*	*Eclipse Dose (cGy)*	*Measured Dose (cGy)*	*Dose %Diff*
		5×5	0.898	0.704	0.948	1.03	293.00	291.55	0.49	180.00	179.96	−0.02
	6	10×10	0.920	0.704	1.000	1.03	266.00	269.77	1.42	180.00	177.58	−1.35
		15×15	0.930	0.711	1.028	1.03	254.00	256.94	1.16	180.00	176.63	−1.88
		20×20	0.936	0.726	1.047	1.03	246.00	245.69	0.13	180.00	176.00	−2.22
15												
		5×5	0.998	0.771	0.926	1.067	240.00	236.81	1.33	180.00	181.71	0.95
	23	10×10	0.993	0.772	1.000	1.067	219.00	220.04	0.47	180.00	177.78	−1.24
		15×15	0.986	0.777	1.040	1.067	211.00	211.55	0.26	180.00	177.58	−1.35
		20×20	0.983	0.790	1.063	1.067	205.00	204.42	0.28	180.00	176.93	−1.71
		5×5	0.898	0.543	0.948	1.03	380.00	377.77	0.59	180.00	179.81	−0.10
	6	10×10	0.920	0.545	1.000	1.03	345.00	348.47	1.01	180.00	176.66	1.86
		15×15	0.930	0.555	1.028	1.03	329.16	330.00	0.66	180.00	175.77	−2.35
		20×20	0.936	0.574	1.047	1.03	313.00	310.75	0.72	180.00	174.67	−2.96
30												
		5×5	0.998	0.628	0.926	1.067	296.00	290.62	1.82	180.00	182.42	1.34
	23	10×10	0.993	0.632	1.000	1.067	269.00	268.79	0.08	180.00	178.68	−0.74
		15×15	0.986	0.643	1.040	1.067	257.00	255.63	0.53	180.00	178.16	−1.03
		20×20	0.983	0.651	1.063	1.067	248.00	248.07	0.03	180.00	177.47	−1.41
		5×5	0.898	0.484	0.948	1.03	422.00	424.22	0.53	180.00	178.81	−0.66
	6	10×10	0.920	0.486	1.000	1.03	391.00	393.20	0.50	180.00	176.71	−1.83
		15×15	0.930	0.489	1.028	1.03	372.00	373.58	0.15	180.00	175.37	−2.57
		20×20	0.936	0.501	1.047	1.03	358.00	356.03	0.27	180.00	174.81	−2.88
45												
		5×5	0.998	0.511	0.926	1.067	362.00	357.07	1.36	180.00	182.83	1.57
	23	10×10	0.993	0.516	1.000	1.067	329.00	329.21	0.06	180.00	179.22	−0.44
		15×15	0.986	0.524	1.040	1.067	313.00	313.69	0.22	180.00	177.66	−1.30
		20×20	0.983	0.536	1.063	1.067	302.00	301.29	0.24	180.00	176.82	−1.77
		5×5	0.898	0.399	0.948	1.03	522.00	514.20	1.49	180.00	179.84	−0.09
	6	10×10	0.920	0.400	1.000	1.03	474.00	474.79	0.17	180.00	176.02	−2.21
60		15×15	0.930	0.406	1.028	1.03	449.00	449.96	0.21	180.00	174.96	−2.81
60												
		5×5	0.998	0.421	0.926	1.067	440.00	433.75	1.42	180.00	182.09	1.16
	23	10×10	0.993	0.429	1.000	1.067	398.00	395.97	0.51	180.00	178.31	−0.95
		15×15	0.986	0.438	1.040	1.067	377.00	375.28	0.46	180.00	176.94	−1.71

### B. Linac Commissioning

In an attempt to measure the effect of using CDMS during a commissioning project, the following parameters have been analyzed, in terms of improvement factor, before and after its implementation:

1. Errors in collected data.
2. Errors in beam modeling.
3. Errors in data book.
4. Completion on time.
5. Clinic's overall satisfaction.

**Figure 10:**
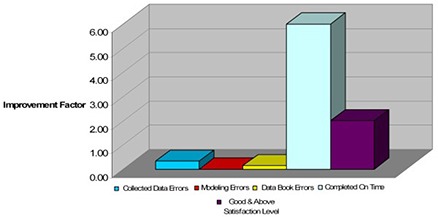
Effect of CDMS clinical implementation on our practice's commissioning process.

A total of 22 commissioning projects were analyzed from the data collection and beam modeling aspects to the clinic's feedback and satisfaction level. Out of the 22, 12 were completed without, and 10 with, the use of CDMS. The results are summarized in Table 3 and Fig. 10, below. The data is presented in terms of improvement factor defined as:

**Table 3:** Effect of CDMS clinical implementation on our practice's commissioning process.

	*Collected Data Errors*	*Modeling Errors*	*Data Book Errors*	*Completed On Time*	*Good & Above Satisfaction Level*
Improvement Factor	0.33	0.0	0.14	6.0	2.0

*QPA and QPP are Quality Parameter After & Prior to implementing CDMS respectively*.

Our results so far have shown than data collection errors were drastically reduced. Beam modeling errors have, so far, been all but eliminated, and the overall satisfaction level of the clinic improved by a factor of 2.

While CDMS was in the development stages, the Radiation Therapy Committee Task Group No.106 of the American Association of Physicists in Medicine published its recommendations for planning, executing and documenting a commissioning task of a linear accelerator.^(^ ^5^ ^)^ Table 4 and Table 5 give a summary of CDMS' compliance with the recommendations of the report.

**Table 4:** CDMS TG‐106 compatibility chart: general data management.

*TG‐106 Recommendation*	*CDMS 1.1 Compliance*
Define the scope of data collection	Yes
Write concise report with all collected data	Yes
Check on the report and collected data	Yes
Backup entire electronic data, analyzed data and spreadsheets	Yes

**Table 5:** CDMS TG106 compatibility chart: commissioning report content.

*TG‐106 Recommendation*	*CDMS 1.1 Compliance*
Formal Commissioning Report, which clearly outlines the scope of the project, what was measured, how, what equipment was used, and the results.	Yes
Open field X‐ray PDD and TMR tables	Yes
Wedged Field X‐ray and TMR tables	PDDs only
X‐ray output factors (Scp, Sc, Sp)	Yes
Field size and depth dependent wedge factor tables	Field size only
Soft wedge (electronic wedge) factor tables	Yes
Transmission factor tables	Yes
Open field off axis tables at selected depths, large field sizes	Yes
Wedge off axis tables at selected depths, largest field size for wedge	Yes, largest square field.
Soft wedge off axis tables at selected depth, largest field size for wedge	No.
Electron cone ratios and effective source distances	Yes
Electron PDD tables	Yes
Provide at least selected isodose curves for reference fields both for electron and photon beams from PDD and profiles	Not Applicable
Printout all scan data	Not Applicable
Compare data from similar machines within your own department or from different institutions; comparison to vendor supplied golden data is also acceptable	Numerical & Graphical
Backup entire electronic data and analyzed data	Yes
Write report	Yes

## IV. CONCLUSIONS

The introduction of CDMS for clinical use appears to have achieved its intended goal of reducing errors in the physics data during the commissioning of linear accelerators. Data collection errors have been drastically reduced, while beam modeling errors were entirely eliminated. CDMS has also significantly improved the confidence of the scanning physicist as well as the satisfaction of the rest of the clinical staff with the progress of the commissioning project. The capacity to computerize many of the common tasks required to gather, process, store, document and access measured data has freed the attention of the commissioning physicist to focus more on the quality of the physics being implemented. In addition, once the commissioning process is complete and the treatment machine has gone clinical, the entire system becomes available for routine physics data query, routine MU calculations, and linac monthly as well as annual calibrations. The physics data, the commissioning and ongoing QA reports can be made readily available electronically to internal reviewers, internal or external auditors and state regulators.

## ACKNOWLEDGEMENTS

I will be forever indebted to Greg Ross, MBA for supporting the development and clinical implementation of CDMS. Also, Marc Sontag, Ph.D., Ron Lalonde, Ph.D., Richard Moreland, Ph.D., for helping with the testing and verification stages. Anu Sharma, MSc., Surendra Rustgi, Ph.D., and Krishna Komanduri, Ph.D., for providing positive feedback. Last, but not least, Evelyn Ruyechan for her role in the administrative use of CDMS.
